# Current Review of Genetically Modified Lactic Acid Bacteria for the Prevention and Treatment of Colitis Using Murine Models

**DOI:** 10.1155/2015/146972

**Published:** 2015-05-04

**Authors:** Alejandra de Moreno de LeBlanc, Silvina del Carmen, Jean-Marc Chatel, Anderson Miyoshi, Vasco Azevedo, Philippe Langella, Luis G. Bermúdez-Humarán, Jean Guy LeBlanc

**Affiliations:** ^1^Centro de Referencia para Lactobacilos (CERELA-CONICET), T4000ILC San Miguel de Tucumán, Argentina; ^2^INRA, Commensal and Probiotics-Host Interactions Laboratory, UMR1319 Micalis, 78350 Jouy-en-Josas, France; ^3^AgroParisTech, UMR1319 Micalis, 78350 Jouy-en-Josas, France; ^4^Federal University of Minas Gerais (UFMG), 31270-901 Belo Horizonte, MG, Brazil

## Abstract

Inflammatory Bowel Diseases (IBD) are disorders of the gastrointestinal tract characterized by recurrent inflammation that requires lifelong treatments. Probiotic microorganisms appear as an alternative for these patients; however, probiotic characteristics are strain dependent and each probiotic needs to be tested to understand the underlining mechanisms involved in their beneficial properties. Genetic modification of lactic acid bacteria (LAB) was also described as a tool for new IBD treatments. The first part of this review shows different genetically modified LAB (GM-LAB) described for IBD treatment since 2000. Then, the two principally studied strategies are discussed (i) GM-LAB producing antioxidant enzymes and (ii) GM-LAB producing the anti-inflammatory cytokine IL-10. Different delivery systems, including protein delivery and DNA delivery, will also be discussed. Studies show the efficacy of GM-LAB (using different expression systems) for the prevention and treatment of IBD, highlighting the importance of the bacterial strain selection (with anti-inflammatory innate properties) as a promising alternative. These microorganisms could be used in the near future for the development of therapeutic products with anti-inflammatory properties that can improve the quality of life of IBD patients.

## 1. Introduction

Inflammatory Bowel Diseases (IBD) describe a group of disorders of the gastrointestinal tract characterized by recurrent inflammation, with periods of relapse and remission, and epithelial injury. Ulcerative colitis (UC) and Crohn's disease (CD) are the two most frequent forms of IBD, clinically characterized by different intestinal location, nature, and the histological features of the inflammatory lesions as well as their association with specific deregulation of the host's immune response. The exact etiology of these pathologies is still unknown; however, it was described that in IBD patients there existed aberrant features of the interaction between intestinal microorganisms and gut immune and epithelial cells, which is manifested as chronic intestinal inflammation [1].

IBD require lifelong treatments, and although they are not generally associated with increased mortality, they can cause significant morbidity. Probiotic microorganisms have appeared as an alternative for IBD patients and their efficiency has been analyzed in experimental animal models and also in clinical trials [2, 3].

This paper will describe some of the mechanisms by which probiotic microorganisms can exert specific benefits against IBD, followed by a revision about the potential use of genetically modify lactic acid bacteria in the prevention and treatment of these recurrent diseases.

### 1.1. Mechanisms Involved in the Anti-Inflammatory Effects of Probiotic Lactic Acid Bacteria

Lactic acid bacteria are the most common microorganisms used as probiotics. Because of specific documented beneficial effects, certain strains of LAB have been designated as probiotics, which have been defined as “live microorganisms that, when administered in adequate amounts, confer a health benefit on the host” [4]. Among these health benefits is the alleviation of IBD symptoms which was reported for some probiotic LAB strains [3].

In this sense, it has been shown that LAB and other probiotic microorganisms can counteract inflammatory processes in the gastrointestinal tract (GIT) through different mechanisms.

One of these is the modulation of the intestinal microbiota related to probiotic administration. It has been shown that* Lactobacillus* (*L*.)* reuteri* could be used to prevent colitis in IL-10^−/−^ mice by increasing the number of lactobacilli in the GIT [5]. In a placebo-controlled trial, the oral administration of* L. salivarius* UCC118 reduced the prevalence of colon cancer and inflammatory activity in the mucosa of IL-10^−/−^ mice by modifying the intestinal microbiota in these animals:* Clostridium *(*C*.)* perfringens*, coliforms, and enterococci counts also decreased significantly in the group treated with the probiotic [6]. Another mechanism by which probiotics can have a positive effect is inhibiting microorganisms through the production of antimicrobial substances such as bacteriocins. Many bacteriocins produced by various species of* Lactobacillus* have been reported [7]. The inhibitory activity of these bacteriocins varies from species to species. It was reported that Lacticin 3147, a broad spectrum bacteriocin produced by a* Lactococcus* (*Lc*.)* lactis*, inhibited a wide range of genetically distinct* C. difficile* strains isolated from healthy subjects and also from patients with IBD [8].

Numerous studies have shown that certain strains of LAB may modulate the host's immune response by regulating the production of cytokines which are involved in regulation, activation, growth, and differentiation of immune cells. One of the ways by which probiotics may exert immunomodulatory activities is by stimulating the production of IL-10 (an anti-inflammatory cytokine). However, not all probiotic strains act in the same way. The anti-inflammatory effects such as the stimulation of IL-10 producing cells are strain-dependent characteristics, and their effectiveness is also dependent on the concentrations used and the method of administration. Thus, the anti-inflammatory effect associated with the administration of a potential probiotic yoghurt to mice was studied in models of acute and chronic intestinal inflammation induced by trinitrobenzene sulfonic acid (TNBS). Animals receiving yoghurt continuously improved immune response with increases in IL-10 and decreases in IL-17 (a proinflammatory cytokine) in the GIT of mice [9, 10]. In another study it was shown that the anti-inflammatory strain* L. salivarius* Ls 33 required the presence of NOD2 receptors to exert its protective effect, which was also related to the local production of IL-10 [11]. By studying the expression of different genes, it was observed that the probiotic strain* L. plantarum* Lp91 caused a significant decrease in the levels of TNF*α* (tumor necrosis factor *α*) and COX-2 (cyclooxygenase-2) in a mouse model of colitis, and this effect was related to significant increases of IL-10 expression [12]. It was also recently observed, through the use of both* in vitro* and* ex vivo* studies, that milk fermented with* L. paracasei* L74 CBA inhibited proinflammatory cytokines without affecting the levels of anti-inflammatory cytokines and that this anti-inflammatory activity depended on metabolic products released during the fermentation process [13]. A new study has also shown that a strain of* Streptococcus* (*S.*)* salivarius* inhibited the activation of the NF-*κ*B* in vitro* by demonstrating anti-inflammatory properties* in vivo* [14].

The improvement of the intestinal barrier function is another mechanism by which probiotic bacteria can benefit the host. The exact mechanism by which probiotics enhance the barrier function and the intestinal mucus is unclear; however, it may be related to alterations in mucus secretion or changes in intercellular interactions of the mucosa and cell stability by modulating the phosphorylation of cytoskeletal proteins and tight junctions [15, 16]. Oral treatment with VSL # 3 (a mixture of eight probiotic bacteria including* Lactobacilli*,* Bifidobacterium*, and* Streptococcus* species) normalized the colonic physiological function and integrity of the mucosal barrier in IL-10^−/−^ mice [15].* L. plantarum* DSM 9843 and* L. reuteri* R2LC also improved barrier function in a model of enterocolitis induced by methotrexate in rats [17]. Some probiotic bacteria can modify the expression of* muc* genes and mucus secretion [18].

Probiotic can also act by reducing oxidative stress, which is characterized by an uncontrolled increase in the concentration of reactive oxidative species (ROS) in the GIT. Thus, another suggested mechanism to prevent inflammation by LAB administration is through the expression of enzymes that are be able to decrease the concentrations of ROS or affect their formation. The levels of these enzymes are often depleted in patients with IBD [19]. Probiotic LAB expressing high levels of antioxidant enzymes could increase these activities in specific locations of GIT and could then contribute to the prevention of oxidative damage, leading to potential applications for the treatment of IBD or posttherapy treatments for IBD or even cancer patients. In this sense,* L. rhamnosus* CNCM I-3690, selected for its antioxidant properties* in vitro*, showed anti-inflammatory activity in a model of colitis* in vivo* [20].

It is important to consider that probiotic characteristics are strain dependent and each probiotic should be tested to know if it has specific beneficial effects, and to describe the mechanism/s involved in their health-promoting properties. In addition, not many mechanisms are usually associated with one individual strain. So, genetic modification of LAB has been also described as a tool to development new treatments for IBD, using microorganisms with GRAS (General Recognized as Safe) status.

## 2. Genetically Modification of Lactic Acid Bacteria for Treatment of IBD

It is theoretically possible, using genetic engineering techniques, to obtain LAB strains that possess a variety of beneficial properties.* L. lactis* is a LAB used in various processes in the food industry and is has been characterized because it does not survive in the digestive tract of animals and humans and thus has the potential to be used without the possibility of survival through the gastrointestinal tract [21].* L. lactis* is normally used as a LAB model because (i) its genome has been completely sequenced, (ii) it is easy to manipulate genetically, and (iii) many genetic tools have already been developed for this species. Based on the identification and isolation of plasmids from native strains of* L. lactis* and other LAB, several cloning vectors have been developed. Using molecular biology techniques, these vectors have been engineered to become important tools for cloning genes of interest, and their products can be controlled with constitutive or inducible promoters.

The development of efficient systems to express genes and suitable protein secretion systems for use in LAB can permit these microorganisms to be used for the production and secretion of a number of heterologous proteins [22]. So, as was explained above, LAB are potential candidates for use as vehicles for the production and delivery of heterologous proteins of technological, medical, or prophylactic interest and several delivery systems are now available for those GRAS microorganisms [23]. The introduction of genes coding for antioxidant enzymes or cytokine production in LAB selected for their probiotic potential, such as the ability to modulate the immune response, may generate very useful strains that can be applied in the treatment of a variety of inflammatory diseases. However, before proposing the genetic modification of anti-inflammatory strains, innate mechanisms of potential vehicle strains should be demonstrated in appropriately designed clinical trials on a large scale. These tests are essential in future studies using genetically modified (GM) strains to demonstrate the differences between wild type and modified microorganisms.

The production of antioxidant enzymes and the production of the anti-inflammatory cytokine IL-10 are two of the most studied anti-inflammatory strategies using GM-LAB and both will be revised in the following sections along with a few examples of other anti-inflammatory compounds that have successfully been produced by GM-LAB.

### 2.1. Lactic Acid Bacteria That Are Genetically Modified to Produce Antioxidant Enzymes

As mentioned previously, in patients with IBD, oxidative stress occurs as the result of an abnormal and recurrent inflammation associated with increased concentrations of ROS. Because few microorganisms produce antioxidant enzymes in the concentrations required to exert biological effects, genetic engineering strategies have been used to obtain more efficient antioxidant producing LAB. Spyropoulos et al. have shown the potential uses of such strains in the treatment of IBD using a variety of animal models [24]. LAB have been used to locally deliver antioxidant enzymes such as superoxide dismutase (SOD) directly in the intestines. This was a major breakthrough because oral administration of SOD is largely limited by its short half-life (5–10 min) in the hostile conditions of the GIT. It has been shown that GM strains of* L. plantarum* and* L. lactis* that are able to produce and release SOD exhibited anti-inflammatory effects in a TNBS induced colitis model [25]. Another experimental study showed the anti-inflammatory activity* L. gasseri* strain producing SOD with an associated reduction of the severity of colitis in IL-10 deficient mice [26].* L. casei* BL23 producing SOD was able to significantly reduce the damage induced by TNBS in mice as shown by increased survival, decreased weight loss, lower microbial translocation to liver, and more importantly a decrease in the damage of the large intestines [27]. These results agree with others presented previously in which the same strain was able to slightly reduce the histological damage degree in a dextran sulphate sodium (DSS) induced colitis model [28].

Because* L. lactis* lacks catalase (as is also the case for most LAB), the heme catalase gene* katE* from* B. subtilis* was added to this industrially important organism resulting in a GM strain able to produce catalase that provided active catalase activities [29]. It was demonstrated that this strain of* L. lactis* producing catalase was able to prevent the development of colon tumors in mice using a chemical induced model [30]. In another study,* L. casei* BL23 was modified to produce heme-independent catalase and this in turn decreased the intestinal inflammatory damage in a TNBS induced mouse model [27]. This result is similar to those obtained previously in which both the native strain* L. casei* BL23 and the derived GM strain producing catalase reduced inflammation degrees in the colon and cecum of mice, using a DSS induced model [31].

*S. thermophilus* CRL807 is a strain that was present in the starter mix, together with 12 other LAB, used to prepare yoghurt with anti-inflammatory and anticancer effects [9, 32]. The anti-inflammatory potential of this strain was demonstrated* in vitro* and* in vivo* [33]. So, the concept that LAB selected for their innate inflammatory potential can be genetically modified to produced antioxidant enzymes and obtain strains with more efficient anti-inflammatory effects was recently evaluated using* S. thermophilus* CRL 807 [33]. Unlike other studies using GM* L. lactis*, it was observed that the unmodified strain exerted anti-inflammatory effects in a TNBS-induced colitis model in mice. It was also observed that both genetically modified* S. thermophilus* CRL 807:CAT and* S. thermophilus* CRL807:SOD (used as suspension or in fermented milks) decreased the severity of inflammation, and these beneficial changes were increased compared to those observed in mice that received the wild type strain. The mixture of both GM-streptococci were also evaluated and shown to exert more anti-inflammatory properties than when each strain was given individually. These results prove that the use of LAB strains that are able to modulate the immune response (innate capacity of* S. thermophilus* CRL 807) and also express antioxidant enzymes show a combined effect and may be a useful strategy in the development of new therapeutics for patients suffering from IBD.

### 2.2. Genetically Modified LAB That Produce the Anti-Inflammatory Cytokine IL-10

Interleukin-10 (IL-10), as explained in the introduction, is one of the major anti-inflammatory cytokines involved in maintaining the homeostasis of the intestinal immune response. It is recognized for its ability to regulate inflammatory responses through the suppression of the proinflammatory cytokine cascades [34], and this is presented as a therapeutic candidate for the treatment of IBD [35]. Furthermore, oral administration of the IL-10 is not a viable option because of its extreme sensitivity to the ambient of the GIT [36]. Although clinical trials conducted to date have shown relatively poor results, the use of new technologies for the delivery of IL-10 at the tissue level has recently been suggested that could become a viable treatment option for certain patients [37]. Thus, different strategies were designed to ensure that IL-10 reaches the GIT, including the use of microencapsulation techniques or viral vectors [38–40]. However, many of these methods are expensive, complicated, or risky methodologically. Therefore, the use of GM-LAB appeared as an attractive alternative for delivering this cytokine at mucosal surface level [22].

The first evidence of GM-LAB as therapeutic vehicle for IL-10 was published in 2000, when it was shown that a strain of* L. lactis* secreting IL-10 prevented the onset of colitis in IL-10^−/−^ mice [41] and reduced inflammation in a model of DSS induced colitis [42]. An important step for the safe use of this GM-LAB for therapeutic purposes in humans was the construction of a biological containment system in a GM strain of* L. lactis* for intestinal delivery of human IL-10 cytokine [43]. In that study, the thymidylate synthase gene of* L. lactis* was replaced by the human IL-10 gene, making this strain unable to grow in the absence of thymidine or thymine. This strain did not contain any antibiotic resistance marker and since thymidine is auxotrophic, the strain could not be spread to the environment making it one of the safest built GM-LAB so far. This containment system was evaluated in patients with CD and was shown not to produce any adverse side effects, and these GM-LAB could only be recovered in feces with the addition of thymidine [44]. However, the clinical outcomes in these patients revealed no statistically significances between those individuals who received the GM-LAB compared with the placebo group. These results showed the need to evaluate new methods of administration to achieve a more effective delivery of IL-10 in the intestinal mucosa using therapeutic LAB [45–49].

*L. lactis* subsp.* lactis* NCDO2118 pXYL:IL-10 is a LAB that produced IL-10 using an expression system that has a food-grade inducer in the xylose-inducible expression system (XIES) [48]. Anti-inflammatory properties were also described for the wild type strain (*L. lactis* subsp.* lactis* NCDO2118) in a DSS induced colitis model in mice [50]. It was demonstrated that milk fermented by* L. lactis* NCDO2118 pXYLCYT:IL-10, strain capable to produce and maintain IL-10 in the bacterial cytoplasm, exerted an anti-inflammatory effect in an acute TNBS induced model of IBD in mice, which was more pronounced than the ones observed with milk fermented by the wild type strain [51]. This effect was related to decreased levels of proinflammatory cytokines in the GIT of mice. The results showed the use of fermented milks as a new form of administration of IL-10 producing* L. lactis*. In this system milk acts as a matrix to protect the bacteria and the cytokine during the passage through the GIT. This new approach could lead to the development of new therapeutic fermented products (functional foods), appropriate for a specific population that suffers gastrointestinal disorders.

*L. lactis* subsp.* cremoris* MG1363 pGroESL:IL-10 produces IL-10 using the protein delivery expression system SICE (Stress-Inducible Controlled Expression) and is based on a stress inducible promoter (pGroESL) that allows the production of the heterologous protein* in situ* (e.g., colon) [45].* L. lactis* capable of delivering the IL-10 protein using the SICE system has the advantage that it does not require an inductor because the adverse conditions of the GIT can by themselves induce this system and all the IL-10 can be locally produced by this LAB in the intestine. Recent studies showed that this strain exerted a protective effect against inflammation in a model of low-grade colon inflammation [52].

Even though protein delivery by GM-LAB showed promising results, DNA delivery by GM-LAB was also evaluated for the production of IL-10 locally by the host's intestinal cells.* L. lactis* subsp.* cremoris* MG1363 pValac:IL-10 is a GM-LAB used for the delivery of IL-10 cDNA. This system is based on a new vector for DNA delivery using lactococci called pValac (vaccination using lactic acid bacteria) that delivers the DNA to the intestinal cells and gives these cells the capacity to produce IL-10 directly at the site of inflammation [46]. It was reported that* L. lactis* MG1363 engineered to express fibronectin binding protein A (FnBPA) was used as a vehicle to deliver the cDNA for IL-10 using the plasmid pValac::*il-10. L. lactis* MG1363 FnBPA + pValac:IL-10 exerted a significant anti-inflammatory effect in a TNBS induced acute model of colitis in mice maintaining high ratios of anti-/proinflammatory cytokines in the intestinal fluids and tissues [53]. The importance of the presence of FnBPA in the GM-LAB was demonstrated using a recombinant strain of* L. lactis* that expresses FnBPA under the control of the nisin inducible expression system [54]; however, the use of the noninvasive strain* L. lactis* subsp.* cremoris* MG1363 pValac:IL-10 was also able to provide the IL-10 cDNA to the host's cells and exerted an in-inflammatory effect in a DSS induced colitis model in mice [55] showing that this DNA delivery system could be used in noninvasive strains.

The effectiveness of both protein and DNA delivery systems was also recently compared using a TNBS induced chronic inflammation model. It was demonstrated that* L. lactis* pValac:IL-10 (noninvasive strain) exerted similar anti-inflammatory effects than* L. lactis* pGroESL:IL-10, when they were administered to the mice during the remission period [56]. Even though the animals that received the strain delivering IL-10 cDNA had higher levels of IL-10 in the intestinal tissues, both systems were effective in maintaining the remission of inflammation which is one of the major problems in current IBD treatments.

### 2.3. Genetically Modified LAB That Produce Other Anti-Inflammatory Compounds

Since TNF is one of the most important proinflammatory cytokines in immune regulated inflammatory processes, the objective of certain groups has been not only to reduce the expression of this cytokine but also to prevent it from being active.* L. lactis* was engineered to secrete monovalent and bivalent murine (m)TNF-neutralizing nanobodies as therapeutic proteins. It was shown that the oral administration of nanobody-secreting* L. lactis* resulted in local delivery of anti-mTNF nanobodies at the colon that significantly reduced inflammation in DSS-induce chronic colitis in mice [57].

Interleukin-27 (IL-27) plays a role in the regulation of T helper (Th) cell differentiation inducing Th1 differentiation and suppressing immune responses. For this reason, it has been proposed that IL-27 could be useful in therapy of diseases mediated by inflammatory cytokines [58]. The 2 genes encoding mouse IL-27 were introduced in* L. lactis* together with a signal sequence which allowed for this cytokine to be secreted. This IL-27 secreting strain was able to reduce colitis, induced via transfer of CD4(+)CD45RB(hi) T cells into Rag(−/−) mice, by increasing the production of IL-10 [59].

Transforming Growth Factor-*β*1 (TGF-*β*) is an inhibitory cytokine recognized as a key regulator of immunological homeostasis and inflammatory responses and was successfully expressed by* L. lactis* and when administered to DSS-induced mice, this GM-LAB decreased colon damage scores [60].

Besides immune regulators, other compounds have also been shown to possess anti-inflammatory potential. The Trefoil Factor (TFF) family of peptides, TFF1 (formerly pS2), TFF2 (formerly spasmolytic peptide, SP), and TFF3 (formerly Intestinal Trefoil Factor, ITF), is involved in the protection of the gastrointestinal tract since they play an essential role in epithelial restitution and repair of mucosal damage [61]. Intragastric administration of TFF-secreting* L. lactis* led to active delivery of TFF at the mucosa of the colon and, in contrast to administration of purified TFF, proved to be very effective in prevention and healing of acute DSS-induced colitis [62]. These same authors then produced a mouth rinse formulation of* L. lactis* secreting human TFF1 which provided a safe and efficient therapeutic tool for treating oral mucositis [61].

It was previously shown that colonic tissues of patients with IBD have increased proteolytic activity and that the use of protease inhibitor Elafin was able to prevent intestinal inflammation in mouse models of colitis [63]. For this reason, serine protease inhibitors such as Elafin and Secretory Leukocyte Protease Inhibitor were expressed in recombinant* L. lactis* and were shown to be very effective anti-inflammatory molecules in a DSS-induced mouse model of colitis [60].

The enzyme 15-lipoxygenase-1 (15-LOX-1) is another molecule that has been proposed as a potential candidate for the resolution of IBD because of its potent anti-inflammatory action. It was shown that the administration of milk fermented by a* L. lactis* strain 15-LOX-1 was effective in the prevention of the intestinal damage associated with inflammatory bowel disease in a TNBS murine model [64].

## 3. Risk Assessment of Genetically Modified Lactic Acid Bacteria with Anti-Inflammatory Properties

Although there is no scientific evidence to support the notion that GM organisms are dangerous for human consumption, it is necessary to show that it is safe to use genetically modified probiotics designed to extend the range of applications covered by natural probiotics.

Consumption of GM microorganisms by human is still a highly controversial issue due to the public perception that genetic manipulation is not “natural.” Scientists need through well-designed studies to report the results for the general population to inform consumers of the benefits that these techniques can confer with minimal risk to the health and the environment, such was the case of the IL-10 producing LAB that was shown to be safe in human clinical trials [44].

## 4. Conclusions

The revision presented here not only showed the potential associated with probiotic microorganisms to be used in patients suffering diseases associated with gastrointestinal inflammation, but also showed the potential use of GM-LAB as new therapies for these patients. It is important to restate the genetic modification of lactic acid bacteria with innate anti-inflammatory properties to produce anti-inflammatory compounds (such as antioxidant enzymes or anti-inflammatory cytokines). It is a promissory tool to obtain new more effective strains with potential applications for IBD patients. These powerful strains should be given as an adjunct treatment to current protocols for IBD patients, and because of the beneficial properties, these could actually improve the quality of life of these patients and aid in preventing the unbalance of beneficial/pathogenic microbiota present in the GIT.
